# Olfactory variation among closely related cactophilic *Drosophila* species

**DOI:** 10.1007/s00359-025-01744-7

**Published:** 2025-06-12

**Authors:** Amber Crowley-Gall, John E. Layne, Byrappa Ammagarahalli, Aaron A. Hamrick, Lucinda P. Lawson, Stephanie M. Rollmann

**Affiliations:** 1 Department of Biological Sciences, University of Cincinnati, PO Box 210006, Cincinnati, OH 45221 USA; 2 Department of Plant Pathology, Entomology and Microbiology, Iowa State University, 1344 Advanced Research and Teaching Building, Ames, IA 50011 USA; 3 Gaiagen Technologies Pvt. Ltd. [Formerly Pest Control (India) Pvt. Ltd.], Bengaluru, 561203 India; 4 Cincinnati Children’s Hospital and Medical Center, Center for Autoimmune Genetics and Etiology, 3333 Burnet Ave, Cincinnati, OH 45229 USA

**Keywords:** Olfaction, Evolution, Host plant, Olfactory sensory neuron

## Abstract

Chemosensation plays an important role in a wide range of behaviors including host identification and localization, oviposition site selection, and mate recognition. Variation in the ability to detect chemical signals may influence behavior in animals like insects that use volatile cues emitted from plants when discriminating between potential hosts. Differences in odor detection has been demonstrated to play a crucial role in driving changes in host use within and between insect species, leading to reproductive isolation between populations and eventual speciation through specialized host adaptation. We examined between-species variation in odor tuning and asked whether it is linked to shifts in host plant use in the *Drosophila repleta* species group, a taxonomic radiation of flies specializing on cacti that exhibits multiple shifts in host plant use across their phylogeny resulting in three current states: (1) *Opuntia* cactus specialists, (2) columnar cactus specialists, and (3) cactus “generalists” which use both hosts. We measured odor response profiles from select olfactory sensillar subtypes across multiple species within the group as well as for the outgroup *D. melanogaster*. Variation in both sensitivity and specificity to odors was observed, with some olfactory sensory neurons exhibiting differences associated with host cactus use. This study is the first in-depth analysis of the olfactory system across the *repleta* species group and provides the opportunity to test for conserved mechanisms in the olfactory system underlying divergence and host shift.

**Supplementary Information:**

The online version contains supplementary material available at 10.1007/s00359-025-01744-7.

## Introduction

For animals that utilize host plants, it is vital to their survival that they select the appropriate one. To accomplish this, they must be able to discriminate the complex sensory signals (e.g. visual, olfactory) they encounter in the environment. For example, herbivorous insects select host plants as feeding and/or breeding substrates using a wide range of sensory cues (Brévault and Quilici 2010; Anderson and Anton 2014; Little et al. 2019). For these animals, chemicals are particularly important cues because they aid in guiding insects to potential attractive hosts across longer distances than visual signals (Aartsma et al. 2017; Webster and Carde 2017; Greenberg et al. 2022). Sensitivity to chemical cues and the specificity with which they stimulate chemoreceptors can have profound effects on species and speciation. Variation in odor sensitivity and specificity across species has been shown to influence behavior and has been proposed to impact divergence within species (Linn et al. 2003; Suinyuy et al. 2015), potentially contributing to speciation (Dekker et al. 2006; Linz et al. 2013; Keesey et al. 2015).

The *Drosophila repleta* species group, which is endemic to North and South America, occupies a wide range of habitats (Oliveira et al. 2012). While several *repleta* species are cosmopolitan the majority of the species group are cactophilic and rely on fermenting host cacti for feeding and breeding (Ruiz and Heed 1988; Ruiz et al. 1990; Oliveira et al. 2012). The *repleta* radiation is estimated to be between 12 and 16 Mya, and the emergence of this cactophilic group coincides with the appearance of *Opuntia*, a flat-leaf cactus, approximately 15 Mya (Oliveira et al. 2012). Basal species within this fruit fly radiation are *Opuntia* specialists (Oliveira et al. 2012). However, usage of this host type is not fixed within this group. There have been at least ten independent transitions within the *repleta* radiation in which species evolved the ability to also use more chemically complex columnar cactus species (becoming cactus generalists) as well as six instances of the evolution of columnar specialists, which lost use of *Opuntia* (Oliveira et al. 2012). Columnar cacti contain elevated levels of secondary metabolites, such as alkaloids and triterpene glycosides (Kircher 1977, 1982; Fogleman et al. 1982; Fogleman and Heed 1989; Fogleman and Abril 1990; Oliveira et al. 2012), which can be toxic to drosophilids. Therefore, flies had to evolve metabolic mechanisms for using these cactus types. For example, cytochrome P450 enzymes have been proposed to act in a detoxification pathway in several species (*D. buzzatii*, *D. koepferae*, *D. mettleri*, *D. mojavensis*, *D. nigrospiracula*, and *D. pachea*), allowing them to use hosts that contain high levels of toxic allelochemicals (Frank and Fogleman 1992; Bono et al. 2008; Carreira et al. 2022). Several other detoxification genes such as *GstD1* and *Adh* have been proposed to play a role in the shift to columnar cactus hosts in populations of *D. mojavensis* (Matzkin 2004, 2008, 2012; Matzkin et al. 2006), *D. buzzatii* (Panis et al. 2016), *D.* *hexastigma* (Lopez-Olmos et al. 2017). These species, and several others, have also exhibited limited detrimental effects of elevated alkaloid concentrations on life history traits (Fogleman et al. 1982; Meyer and Fogleman 1987; Soto et al. 2012, 2014; Padro et al. 2014, 2018).

Attraction to host substrates in drosophilids is mediated in large part by their ability to detect host volatile cues using their olfactory systems. The *Drosophila* olfactory system has been widely studied through the traditional genetic model *D. melanogaster* and comprises two olfactory organs, the antenna and the maxillary palp. These olfactory organs are covered in specialized hair-like structures called sensilla, which can be categorized into distinct types by their size, morphology, and the types of volatile cues they detect: basiconic, coeloconic, and trichoid (Shanbhag et al. 1999; Vosshall et al. 1999; Yao et al. 2005; Kurtovic et al. 2007; van der Goes van Naters and Carlson 2007; Vosshall and Stocker 2007). All three types of sensilla can be found on the surface of the antenna (Shanbhag et al. 1999; Vosshall and Stocker 2007). Each sensillum typically contains the dendrites of two olfactory sensory neurons (OSNs), which can be defined by their unique odor response profiles (Vosshall and Stocker 2007).

Variation in neurophysiological responses in OSNs has been shown to be associated with alterations in amino acid sequence (Prieto-Godino et al. 2017) or expression levels (Crowley-Gall et al. 2016) of odorant receptors, as well as by odorant receptor gene loss (de Bruyne et al. 2010). Therefore, electrophysiological studies can be useful in screening for underlying genetic variation between populations and/or species. Such differences have been previously examined across drosophilid species but these have focused on members of the Sophophora subgenus, while members of the Drosophila subgenus, which contains the *repleta* species group, are typically used as an outgroup (Stensmyr et al. 2003a; de Bruyne et al. 2010). To date within the *repleta* group, genome sequences have been published for *D. arizonae*, *D. aldrichi*, *D. antonietae*, *D. borborema*, *D. buzzatii*, *D. hydei*, *D. koepferae*, *D. mercatorum*, *D. mojavensis*, and *D. navojoa* (Guo and Kim 2007; Drosophila 12 Genomes Consortium 2007; Sanchez-Flores et al. 2016; Rane et al. 2019; Li et al. 2021; Benowitz et al. 2024; Moreyra et al. 2023; *Drosophila buzzatii* Genome Project), but *D. mojavensis* is the only species that has had its olfactory system extensively examined (Date et al. 2013, 2017; Crowley-Gall et al. 2016, 2019; Nemeth et al. 2018; Ammagarahalli et al. 2021; Depetris-Chauvin et al. 2023). *Drosophila mojavensis* has four populations, endemic to parts of southwestern United States and northwestern Mexico. Three populations utilize columnar cactus species and one is an *Opuntia* specialist (Etges and Heed 1987). *Opuntia* and columnar cacti have distinct volatile profiles, and OSN responses between *D. mojavensis* populations vary with shifts in host plant use (Date et al. 2013, 2017; Crowley-Gall et al. 2016). The observed associations in host use and olfaction response in *D. mojavensis* and the repeated instances of columnar specialization across the *repleta* species group suggest that this relatively understudied group is a valuable model for examining variation in olfactory responses as a result of host shifts in a phylogenetic framework. This group provides the opportunity to assess the extent to which variation in OSN responses are repeatedly associated with shifts from the ancestral *Opuntia* host to more chemically complex columnar cacti.

**Fig. 1:**
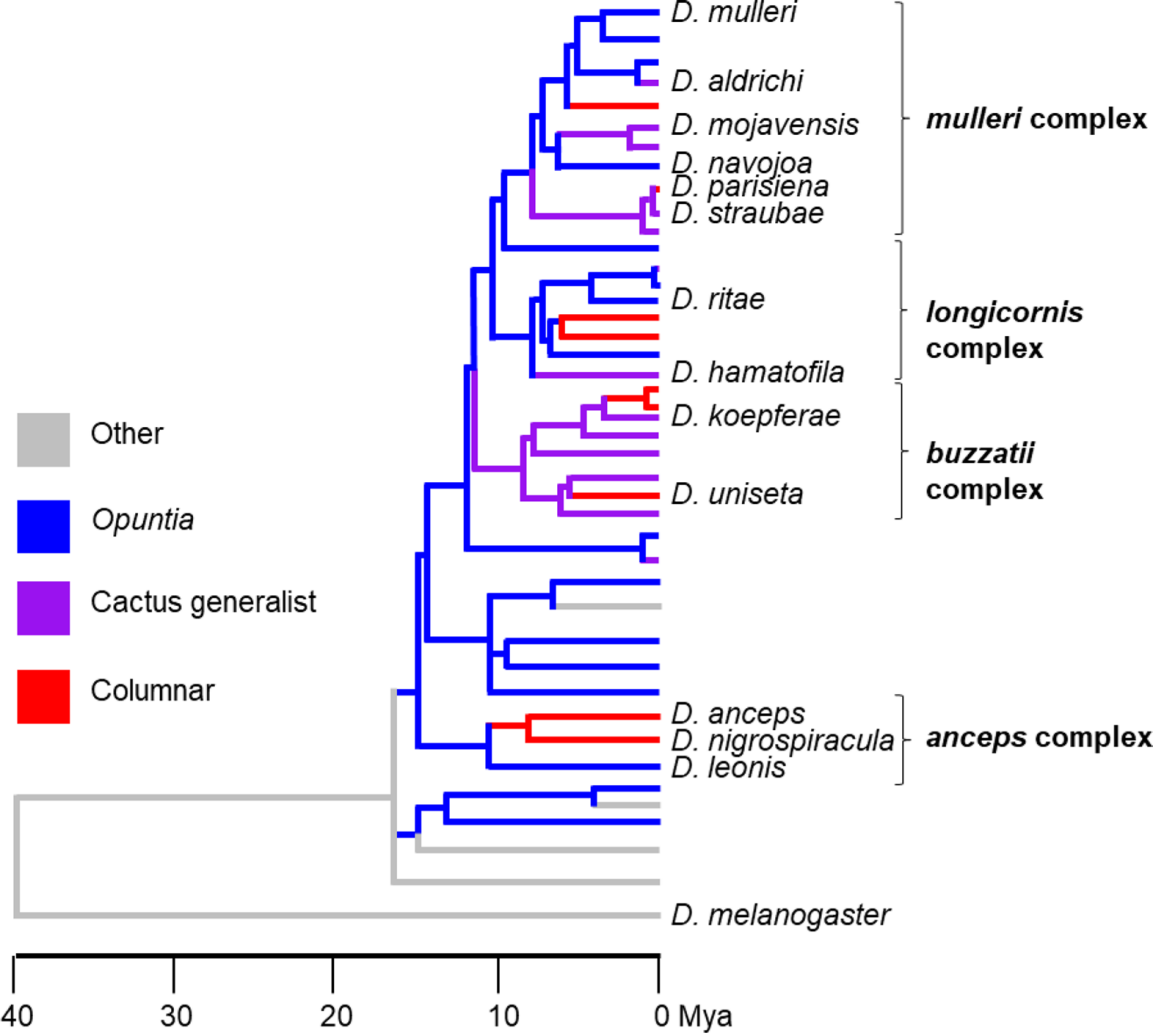
Phylogeny of the *Drosophila repleta* species group with *Drosophila melanogaster* as an outgroup [adapted from Oliveira et al. 2012]. The 13 cactophilic species chosen for this study are labelled on the phylogeny and represent members of the *mulleri* (*D. mulleri*, *D. aldrichi*, *D. mojavensis*, *D. navojoa*, *D. parisiena*, and *D. straubae*), *longicornis* (*D. ritae* and *D. hamatofila*), *buzzatii* (*D. koepferae* and *D. uniseta*) and *anceps* (*D. anceps*, *D. nigrospiracula* and *D. leonis*) complexes.

This study examined the extent to which odor tuning has diverged as a result of the multiple host shifts from *Opuntia* cactus to more chemically complex (and toxic) columnar cacti within the *repleta* group. Neurophysiological responses of two sensillar subtypes were recorded across 13 *repleta* group species as well as *D. melanogaster* to a suite of odors diagnostic of the sensillar subtypes and representative of common cactus volatile cues. These 13 cactophilic species are from four different species complexes within the *repleta* group (*mulleri*, *longicornis*, *buzzatii* and *anceps*) ensuring coverage of all complexes within the species group that include a transition to columnar specialization and representation of *Opuntia* specialists, columnar specialists, and cactus generalists (Fig. 1, adapted from Oliveira et al. 2012). Specifically, we recorded from the antennal basiconic (ab) sensillar subtypes 2 and 3 (ab2 and ab3) to identify any species-specific responses to olfactory cues. The ab2 subtype contains OSNs that display odor response profiles that are both conserved (ab2A) and divergent (ab2B) across previously examined drosophilid species (Stensmyr et al. 2003a; de Bruyne et al. 2010; Mansourian and Stensmyr 2015). The OSNs in the ab3 subtype have not only shown highly divergent odor tuning responses across drosophilid species, but one (ab3A) has also been proposed to be adapted for species-specific host detection in several species, such as *D. sechellia*, *D. erecta*, and *D. suzukii* (Dekker et al. 2006; Linz et al. 2013; Keesey et al. 2015; Mansourian and Stensmyr 2015). Therefore, an examination of these two sensillar subtypes (4 OSNs) provides an opportunity to assess whether comparable levels of odor tuning, conservation and divergence, are present in the *repleta* group as well as to examine if OSN ab3A plays a role in host shift. Overall, comparing neurophysiological responses across a phylogeny can provide insight into how variation in the olfactory system contributes to the process of divergence and host specialization and if shifts in host use are facilitated in the same way each time, or if novel changes underlie each host-shifting event.

## Methods

### *Drosophila* stocks

Flies were reared on a 12:12 L/D cycle at 25^o^ C on either standard cornmeal (*D*. *melanogaster*), potato (*D*. *anceps* and *D*. *nirgrospiracula*) or banana-cactus media. Fly lines were obtained from the Bloomington *Drosophila* Stock Center (Indiana University, Bloomington, IN), *Drosophila* Species Stock Center (Cornell University, Ithaca, NY) or kindly provided by Dr. William Etges, Dr. Alfredo Ruiz or Dr. Michal Polak and are as follows: *D. melanogaster* (64349), *D. mulleri* (15081-1371.01), *D. aldrichi* (15081-1251.22), *D. mojavensis* (SQ59a; San Quintin, Baja California, Mexico), *D. navojoa* (15081-1374.11), *D. parisiena* (15081 − 1392.00), *D. straubae* (15081-1462.03), *D. ritae* (15081-1471.03), *D. hamatofila* (15081-1301.07), *D. koepferae* (15081-1305.01), *D. uniseta* (UN-7; Salamanca, Colombia), *D. anceps* (15081 − 1261.10), *D. nigrospiracula* (Superstition Mt Wilderness 2018), and *D. leonis* (15081-1395.01).

### Odor set

Chemical compounds were obtained at the highest level of purity available from Sigma-Aldrich (St. Louis, MO). Selected compounds include volatiles found in several columnar and *Opuntia* species identified through studies of the volatile composition of either natural rots or laboratory inoculated rots (Flath and Takahasi 1978; Ammar et al. 2012; Date et al. 2013, 2017; Wright and Setzer 2013a, b, 2014a, b; Supplementary Table 1). Additionally, odorants that are diagnostic for ab2 and ab3 sensillar subtypes, based on previous work were included (de Bruyne et al. 2001; Bruyne et al. 2010; Stensmyr et al. 2003a; Hallem and Carlson 2004, 2006; Hallem et al. 2004; Crowley-Gall et al. 2016). Compounds were diluted in either paraffin oil or water depending on solubility.

### Single sensillum recordings

We characterized the odor response profile of the ab2 and ab3 sensillar subtype to a suite of odorants for 13 species in the *repleta* group, as well as the outgroup *Drosophila melanogaster* using single sensillum recordings. These 13 species reflect the different host cacti use within the *repleta* group and are spread across multiple species complexes, covering several independent shifts to columnar cactus use. Out of the 13 species, four were *Opuntia* specialists (*D. mulleri*, *D. navojoa*, *D. ritae* and D. *leonis*), five were cactus generalists (*D. aldrichi*, *D. mojavensis*, *D. straubae*, *D. hamatofila* and *D. koepferae*), and four were columnar specialists (*D. parisiena*, *D. uniseta*, *D. anceps*, and *D. nigrospiracula*) (Fig. 1).

Single sensillum recordings (SSRs) of the antenna were performed as previously described (de Bruyne et al. 2001; Crowley-Gall et al. 2016). Briefly, large basiconic sensilla were viewed under a compound light microscope and a tungsten recording electrode was inserted into the base of a sensillum and a reference electrode was inserted into the eye of the fly. Odorants (1%) were introduced to the fly for 0.5 s via a glass pipet into a continuous stream of humidified air, and a minimum of 30s between stimulus deliveries. Signals were amplified, digitized and recorded using an IDAC4 and AUTOSPIKE software (Syntech, Buchenbach, Germany). The difference in the number of action potentials counted in 0.5s interval pre- and post-stimulation was considered the response of individual neurons. A minimum of 5 recordings, from 5 flies, were obtained for each odorant for each sensillar subtype, except for *D. straubae* ab3 (*n* = 3), *D. leonis* ab3 (*n* = 4), and *D. nigrospiracula* ab3 (*n* = 1). The *D. nigrospiracula* ab3 recordings were not easily identifiable and were excluded from cross species comparisons. Additionally, the ab3 sensillar subtype was not detected in *D. parisiena* and *D. anceps* in this study.

### Data analysis

All statistical analyses were performed using R v.3.6.2 (R Core Team 2019). Differences in responses to single odorants were analyzed for all ‘responding odorants’ (odorants eliciting a response above 30 spikes/second) with an ANOVA followed by a Tukey-Kramer post hoc test (packages “stats”, “emmeans” and “multcomp”; Hothorn et al. 2008; R Core Team 2019; Lenth 2022). Results were corrected for multiple testing using a false discovery rate of 0.05 (package “stats”; Benjamini and Hochberg 1995; R Core Team 2019). Overall similarities in response patterns across species were characterized with a hierarchical cluster analysis with Ward’s method on the mean response to all odorants using (packages “factoextra” and “cluster”; Maechler et al. 2019; Kassambara and Mundt 2020). A second cluster analysis, which accounted for variation in sensitivity between species, was performed on mean values normalized to the highest mean response within each species.

Phylogenetic analyses of trait variation were run on the ‘responding odorants’ data and phylogenetic data from Oliveira et al. (2012) using a variety of packages in R (ape, diversitree, phytools, geiger, ggplot2, nlme, vegan, mvMORPH; Harmon et al. 2008; Revell 2011; FitzJohn 2012; Oksanen et al. 2013; Clavel et al. 2015; Wickham 2016; Pinheiro et al. 2017; Paradis and Schliep 2019). This determined whether distantly related species with the same host type have convergent selective pressures towards similar odorant response profiles. In order to limit odorants to putatively meaningful compounds, a phylogenetic principal component analysis (pPCA) was completed for each neuron independently (ab2A, ab2B, ab3A, ab3B), which enabled us to detect separation between any groups (*Opuntia* specialists, columnar specialists, cactus generalists). The top contributing odorants were identified from each PC with separation. When pPCA analyses showed clustering based on host-type, further tests were run to determine whether one primary odorant underlay a convergent response based on host-type or if similarities might be by chance or by weak effect from multiple odorants together. For these tests, first a list of the top 10 pPCA odorants was created from the highest contributors to separation in any pPCA analysis as well as odorants that contributed across multiple neurons. To then test whether odorant responses were convergent across lineages based on their host (columnar specialist, *Opuntia* specialist, cactus generalists) or if a single or multiple odorants dominated the pPCA separation based on host-type, models of Brownian motion (single rate and multiple rate), λ = 1 and 0, and Ornstein-Uhlenbeck models (single optima and multi-optima) were compared for each odorant identified in the pPCA analyses on a phylogenetic tree of taxa used in this study (pruned from Oliveira et al. 2012).

## Results

### OSN responses across 14 species

#### The ab2 subtype

**Fig. 2:**
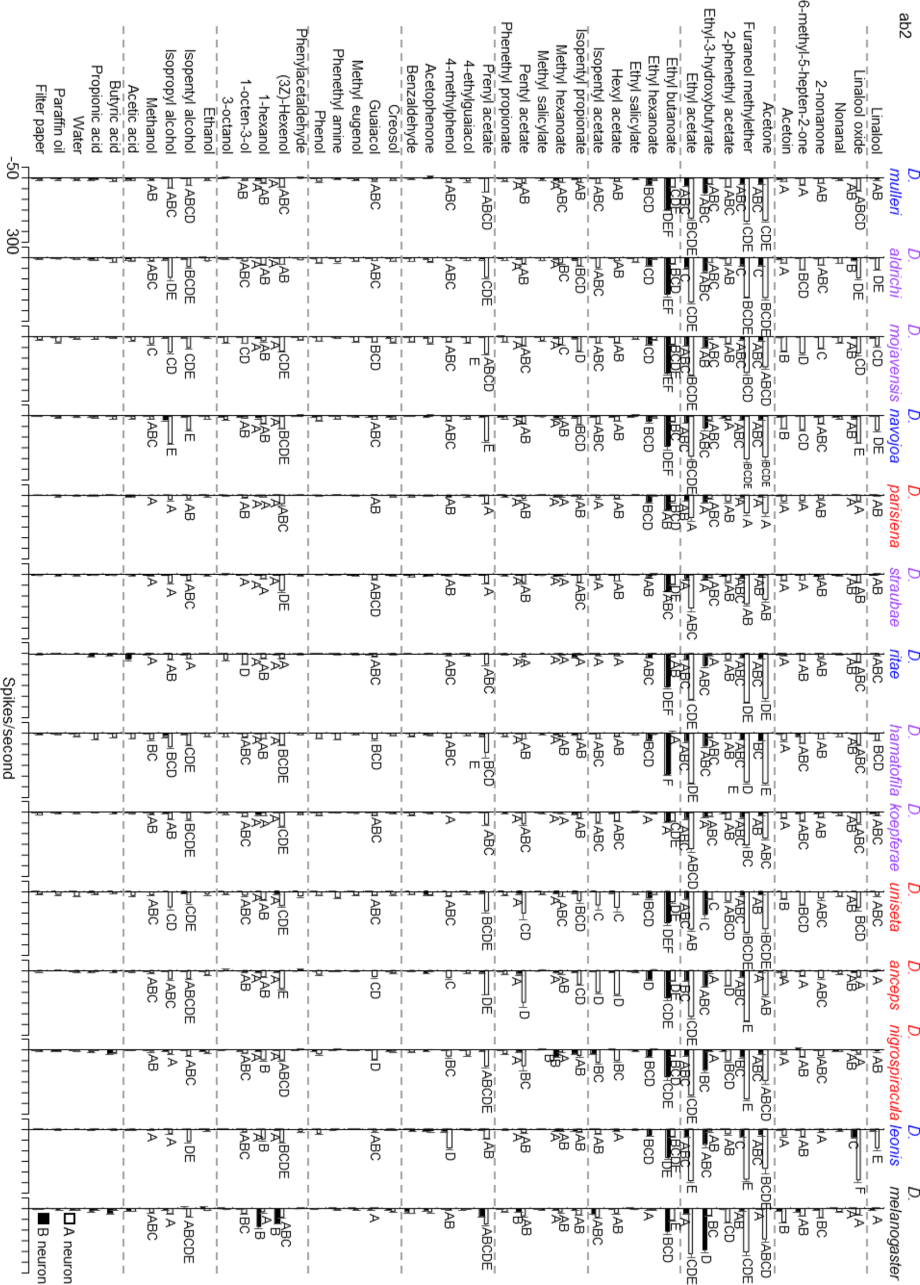
OSN response profiles of the ab2 sensillar type of 14 drosophilid species to a suite of 43 odorants as well as blank (filter paper) and vehicle (paraffin oil and water) controls. Responses from the A neuron are represented by empty bars, while B neuron responses are shown by filled in bars. Significant differences in electrophysiological responses between species to odors eliciting a response greater 30 spikes/second are shown by different letters. Cactus host associations are indicated by color of species name: *Opuntia* specialists (*D. mulleri*, D. *navojoa*, *D. ritae*, *D. leonis*) are shown in blue, cactus generalists (*D. aldrichi*, *D. mojavensis*, *D. straubae*, *D. hamatofila*, *D. k.oepferae*) are shown in purple, and columnar specialists (*D. parisiena*, *D. uniseta*, *D. anceps*, *D. nigrospiracula*) are shown in red

The ab2 sensillar type was identified in all 14 species and the response profile was generally conserved, with a few notable exceptions (Fig. 2). Responses of the ab2B neuron differed between the *repleta* group and *D. melanogaster*. For instance, the *repleta* group shows a reduction in sensitivity of this neuron to 1-hexanol, (3Z)-Hexenol and ethyl-3hydroxybutyrate compared to *D. melanogaster*. Responses of the ab2A neuron showed several notable differences within the *repleta* group. For instance, *D. leonis* showed increased sensitivity in responses to linalool, linalool oxide and 4-methyl phenol when compared with the other species examined. In particular this shift in odor response highlights a change in odor specificity within the *anceps* complex as all three odors are strong responders in *D. leonis* but linalool and linalool oxide are considered ‘non-responding odorants’ in the other members of the *anceps* complex examined, *D. anceps* and *D. nigrospiracula*, and while 4-methylphenol is a ‘non-responding odorant’ in *D. anceps* it just crosses the threshold of being a responding odorant in *D. nigrospiracula*.

#### The ab3 subtype

**Fig. 3:**
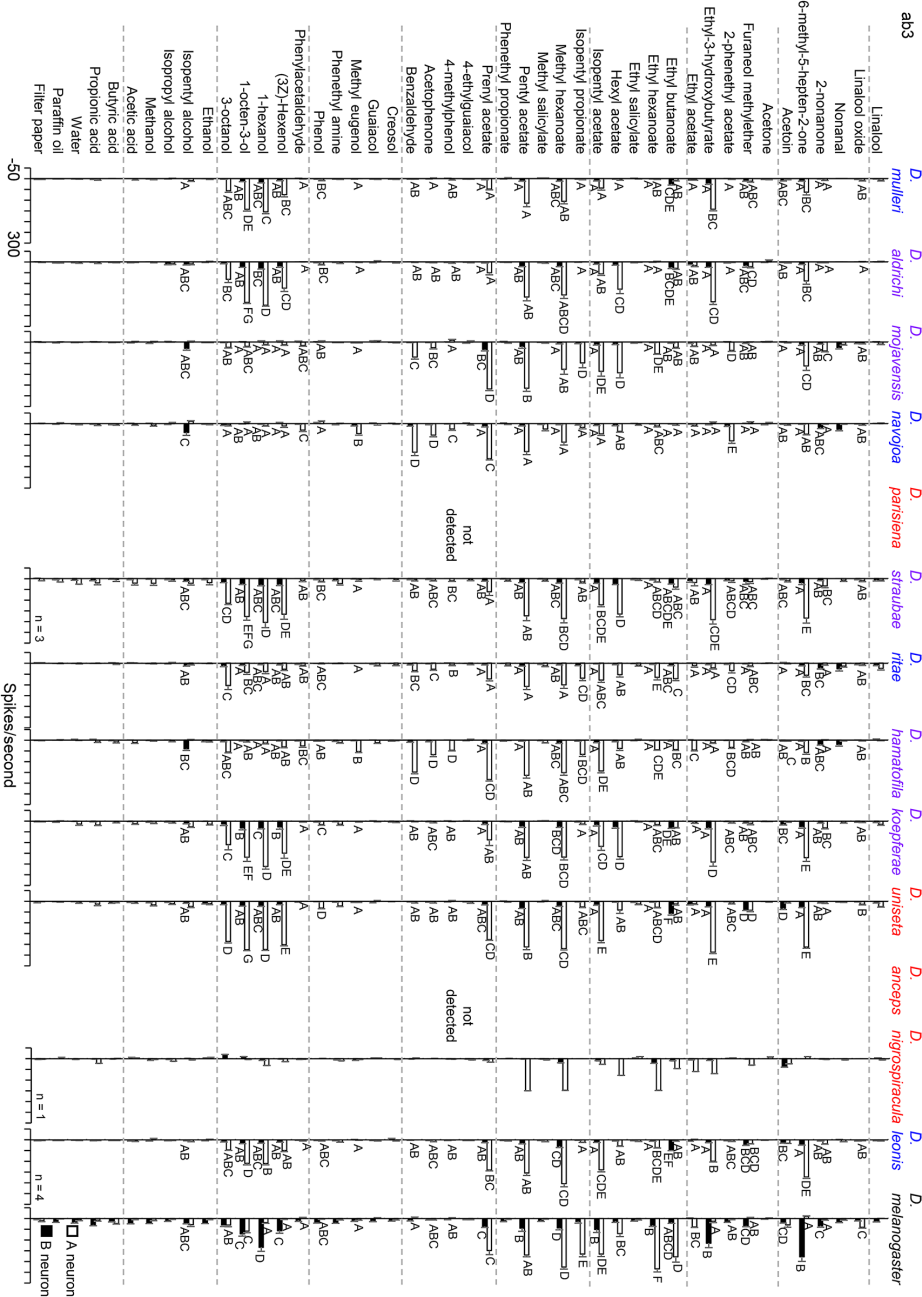
OSN response profiles of the ab3 sensillar type of 11 drosophilid species to a suite of 43 odorants as well as blank and solvent (paraffin oil and water) controls. The ab3 sensillar type was not detected in *D. parisiena* and *D. anceps*. Limited ab3 recordings were made from *D. nigrospiracula* (*n* = 1) and were not included in cross species analysis. Responses from the A neuron are represented by empty bars, while B neuron responses are shown by filled in bars. Significant differences in electrophysiological response between species to odors eliciting a response greater than 30 spikes/second are shown by different letters. Cactus host associations are indicated by color of species name: *Opuntia* specialists (*D. mulleri*, D. *navojoa*, *D. ritae*, *D. leonis*) are shown in blue, cactus generalists (*D. aldrichi*, *D. mojavensis*, *D. straubae*, *D. hamatofila*, *D. koepferae*) are shown in purple, and columnar specialists (*D. parisiena*, *D. uniseta*, *D. anceps*, *D. nigrospiracula*) are shown in red.

The ab3 sensillar type was identified in 11 of the 14 species examined, with high levels of variation observed (Fig. 3). In this study *D. melanogaster* ab3B neuron has sensitivity to 6-methyl-5-hepten-2-one, ethyl-3-hydroxybutyrate, (3Z)-Hexenol, 1-hexanol and 1-octen-3-ol, and the ab3A neuron sensitivity to ethyl butanoate and ethyl hexanoate, all of which appear to be severely reduced or lost in the *repleta* group. We were unable to detect an ab3 sensillar type in *D. parisiena* and *D. anceps*. Additionally, we only recorded one ab3-like sensillum in *D. nigrospiracula* and due to limited data, this recording was not included in further cross species analyses.

### Clustering of odor response profiles

**Fig. 4:**
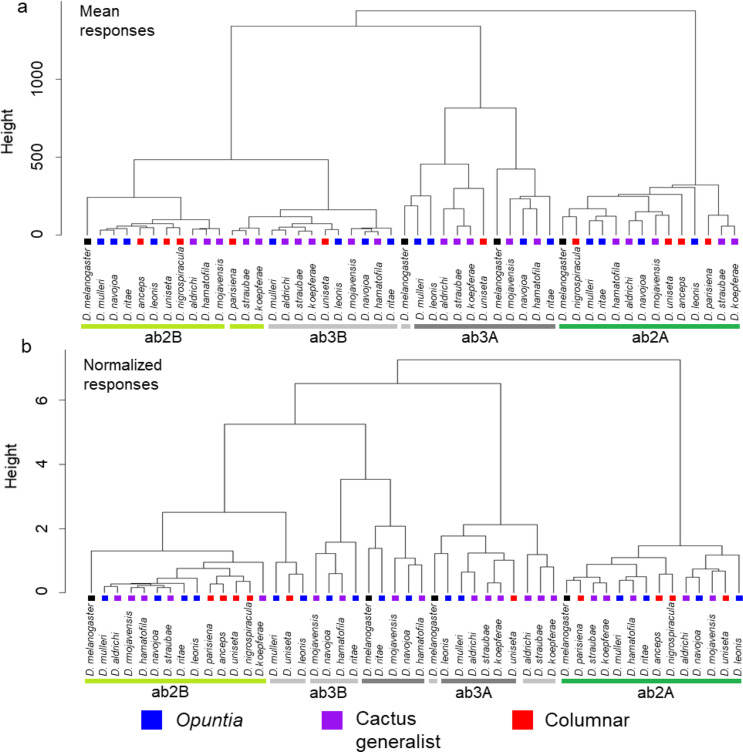
Cluster analysis of mean (**a**) and normalized mean (**b**) responses of both the A and B neuron for ab2 [14 species] and ab3 [11 species] sensilla to the suite of tested odorants

When the mean odor response profiles for each neuronal type in ab2 and ab3 sensilla for all recorded species were subjected to a hierarchical cluster analysis, the four neuronal types clustered into four corresponding branches, with a few exceptions (Fig. 4a). The ab2A and ab3A neuronal responses for all *repleta* group species and the *D. melanogaster* outgroup formed distinct clusters, but this was not the case for the ab2B and ab3B neuronal types. The *D. melanogaster* ab3B clustered more closely with *repleta* group ab3As than ab3Bs, while *D. parisiena*, *D. straubae* and *D. koepferae* ab2B clustered with ab3B. The former result appears to be due to the increased sensitivity of *D. melanogaster*, noted above, to several odorants noted. In the latter case, these three species show a general reduction in sensitivity to ethyl butanoate and ethyl-3-hydroxybutyrate, which were the only odors consistently eliciting high OSN responses for the ab2B neuron in the other *repleta* species (Fig. 2). Their ab3B were positively identified as such by their co-occurrence with clearly identifiable ab3A, despite somewhat resembling ab2B. Because these cases of decreased overall sensitivity in a subset of species may mask shifts in sensitivity to select odorants, we performed another cluster analysis after normalizing mean responses to the highest mean response within each species. This focuses the analysis on the relative sensitivity to odorants, by minimizing the effect of absolute sensitivity. (Fig. 4b). In this normalized cluster all of the ab2A and ab2B neuronal responses cluster together by type and notably the ab2B responses of the four species specializing on columnar cactus (*D. parisiena*,* D. uniseta*, *D. anceps* and *D. nigrospiracula)* form a distinct cluster. Additionally, the ab2B neuronal responses of all members of the more recently diverged *mulleri* and *longicornis* complexes cluster together, with the exception of *D. parisiena* which is a part of the previously mentioned columnar cactus cluster. The responses of the ab3A and ab3B neuron show greater separation than was seen in the non-normalized cluster. Even though the two groups formed by the ab3A neuronal responses separate into different clusters, the species within each group remains the same as in the non-normalized cluster. This separation appears to be driven by a reduced response to (3Z)-Hexenol, 1-hexanol, 1-octen-3-ol, 3-octanol and ethyl-3-hydroxybutyrate in *D. melanogaster*, *D. ritae*, *D. mojavensis*, *D. navojoa* and *D. hamatofila* relative to the species in the other group (Fig. 3). Interestingly, there were no clear associations with host use or phylogeny as the two *longicornis* complex species fall into one group, while the two *buzzatii* complex species are in the other, and the *mulleri* complex has species in both groups. Additionally, the ab3B neuronal response profiles are separated into four groups, one of which is *D. melanogaster* whose separation was previously noted in the non-normalized cluster, while the remaining three groups’ separation appears to be driven by weak responses to several odorants: (1) *D. mulleri*, *D. unise*ta and *D. leonis* (furaneol methylether and ethyl butanoate); (2) *D. mojavensis*, *D. navojoa*, *D. hamatofila* and *D. ritae* (nonanal and isopentyl alcohol); (3) *D. aldrichi*, *D. straubae* and *D. koepferae* ((3Z)-Hexenol, 1-hexanol, and 1-octen-3-ol) (Fig. 3). Again, like in the ab3A neuronal responses there is no clear association with phylogeny or host use in the groupings of ab3B responses. In summary, the ab2A neuronal responses showed high levels of conservation. The ab2B responses exhibited separation associated with some cases of reduced sensitivity, which, once accounted for by normalizing the responses, revealed one distinct cluster with a unique grouping of columnar cactus specialists. Finally, higher levels of divergence were observed in both ab3A and ab3B responses, particularly after normalization, and a small number of odorants were identified that could be driving separation of neuronal responses.

### Phylogenetic analyses of odorant response evolution

**Fig. 5:**
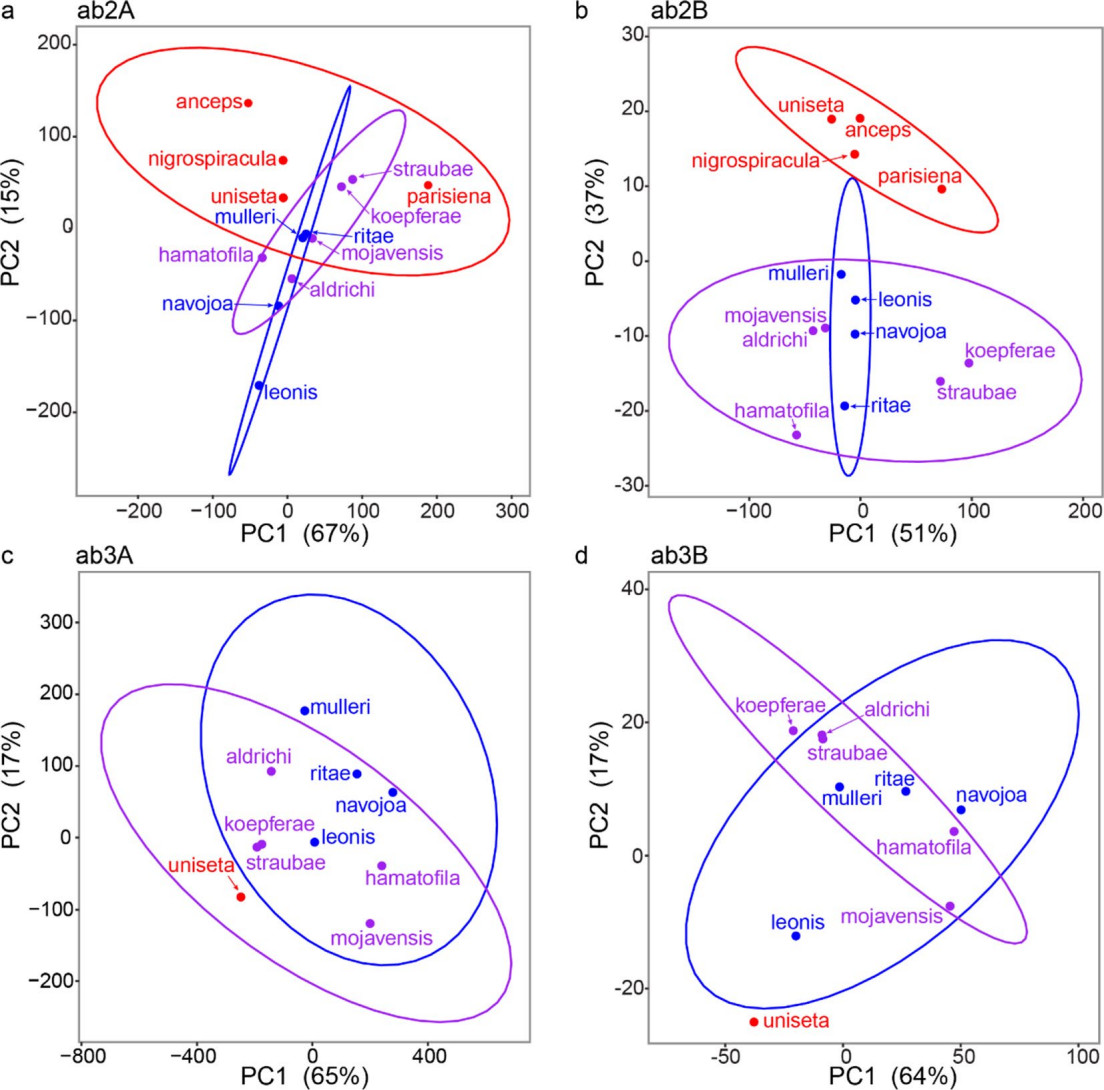
Phylogenetic principal component analysis for odorant responses of each neuron. *Opuntia* specialists (*D. mulleri*, D. *navojoa*, *D. ritae*, *D. leonis*) are shown in blue, cactus generalists (*D. aldrichi*, *D. mojavensis*, *D. straubae*, *D. hamatofila*, *D. koepferae*) are shown in purple, and columnar specialists (*D. parisiena*, *D. uniseta*, *D. anceps*, *D. nigrospiracula*) are shown in red. 90% conficence intervals are shown as calculated through the stat_ellipse function in ggplot2. Proportion of variance for each PC is shown.

Phylogenetic PCA (pPCA) analyses were used to determine if all species of a specific host type (e.g. columnar specialists) had similar odorant sensitivity profiles despite independent phylogenetic origins. Overall, we found general overlap of the *Opuntia* specialists with the cactus generalist species, and separation from columnar specialists (Fig. 5). Specifically, ab2A showed modest separation along PC2 between columnar (red) and other host types (though not significant at the 90% confidence interval level, Fig. 5a), and ab2B showed clear separation along PC2 (90% confidence intervals do not overlap (Fig. 5b)). The ab3 analyses included only one columnar specialist, which made interpretations more difficult. However, that species (*D. uniseta*) occupied its own space in PC1 and PC2 for both ab3A and ab3B and was outside of the 90% confidence intervals for ab3B (Fig. 5d), suggesting that odor response profiles may be quite different in species using columnar cactus. Additional sampling of species for all four neurons are necessary to have sufficient statistical power to detect host-specific patterns.

To determine if these different response profiles were dominated by a single-odorant shift or general shifts in chemical sensitivity, a list of “top candidates” were identified for further analyses. First the odorants which had the highest contributions to the previously mentioned differentiating axes (ab2A- PC2; ab2B- PC2; ab3A- PC1 and PC2; ab3B- PC1 and PC2) were identified. Additionally, odorants which had high loading values and were also shared across neuronal types (e.g., ab2A and ab3B) were identified. This list was: ab2A: linalool oxide, 2-phenethyl acetate, linalool, and 4-methylphenol; ab2B: ethyl hexanoate, furaneol methylether, and ethyl acetate; ab3A: 1-octen-3-ol, 1-hexanol, and ethyl-3-hydroxybutyrate; ab3B: 1-octen-3-ol, 1-hexanol, furaneol methylether and ethyl-3-hydroxybutyrate.

To test for a signal of selection on each of these odorant signals within a phylogenetic framework, each was compared to models of trait selection impacted by host-type (i.e., single or multiple optima of odorant response rate based on host type) and neutral variation among the phylogenetic tree for each receptor. For all analyses except for ab3B furaneol methylether and ab2A 2-phenethyl acetate, Brownian motion (bm1 and bmm) was the best-fitting model according to the Akaike information criterion, implying that there is no selective pressure towards different response rates based on host type for any of the odorants identified in the pPCA (Supplementary Table 2). Responses to furaneol methylether in ab3B and 2-phenethyl acetate in ab2A both did not differentiate strongly between the two best models for each (Δ < 2), though likelihood ratio tests of Brownian motion models (bm1 vs. bmm) and OU models (ou1 vs. oum) showed that the multiple rate models were better than their single rate counterparts. In both cases, the *Opuntia* specialists and the generalist had similar estimates of theta, and the columnar specialists were different (e.g., 10.3 and 8.1 respectively for *Opuntia* and generalists, and 46. 1 for columnar in furaneol methylether. Likewise, 30.6 and 25.7 compared to 62.6 in 2-phenethyl acetate). However, the low ΔAIC score between Brownian (random) and OU (selection) models for both odorants means that these different response rates are unlikely to be biologically meaningful. Furaneol methylether is considered a diagnostic odorant and to date has not been found to be ecologically relevant in cactophilic flies (Dweck et al. 2016). While 2-phenethyl acetate has been found in the volatile headspace of laboratory rots of cacti, its presence has been found to vary based on what microbes are inducing rot (Date et al. 2017) and it has been found in the volatile profile of both columnar and *Opuntia* cacti (Date et al. 2013). This suggests that it likely does not play a role in host shifts between cactus types within this system. The analyses collectively show that while an overlap of *Opuntia* specialist and generalist species was observed along with a separation of columnar specialist odorant response profiles, this does not appear to be achieved by re-evolving different response rates to a single odorant.

## Discussion

Organisms encounter and process complex chemical signals while navigating their environments in search of a suitable host plant. Examining differences in olfactory sensitivity provides insight into the evolution of sensory mechanisms enabling or driving host specialization. Recent work on gall midges (Molnar et al. 2018) used a phylogenetic framework to identify a relationship between olfactory responses and perennial hosts revealing clustered olfactory responses in two species associated with grasses as well as two Brassicaceae specialists despite their phylogenetic separation. However, these results used a group of insects with multiple ecologies and host association and phylogenetic frameworks using species that re-evolve categorical ecological traits provide a powerful tool to examine conserved mechanisms of divergence.

In this study we examined the olfactory system of the *repleta* species group. The majority of the work on species in the *repleta* group has focused on examining reproductive isolation, transcriptome analysis, nutritional utilization/detoxification and life history traits (e.g., Ganter and Starmer 1989; Massie and Markow 2005; Matzkin 2004; Matzkin et al. 2006; Hasson et al. 2008; Matzkin 2008; Bono and Markow 2009; Jennings and Etges 2010; Castrezana and Bono 2012; Soto et al. 2012; Bono et al. 2014; Guillen et al. 2014; Soto et al. 2014; Lopez-Olmos et al. 2017). To date, *D. mojavensis* is the only species within the group in which the olfactory system has been extensively studied (Date et al. 2013, 2017; Crowley-Gall et al. 2016; Crowley-Gall et al. 2019; Nemeth et al. 2018; Ammagarahalli et al. 2021; Depetris-Chauvin et al. 2023) and differences in odor sensitivity and specificity have been seen between populations of a single species specializing on either columnar (barrel cactus, *Ferocactus cylindraceus*) or *Opuntia* cactus (Crowley-Gall et al. 2016). This study is a first examination of the olfactory system across the *repleta* group through a characterization of the ab2 and ab3 sensillar types.

The ab2 and ab3 sensillar types were chosen due to their ability to be identified as large basiconics, and because they both are hypothesized to contain neurons that are highly variable in their odor response profiles across previously studied drosophilids (de Bruyne et al. 2001; Bruyne et al. 2010; Stensmyr et al. 2003a; Hallem et al. 2004; Stokl et al. 2010; Mansourian and Stensmyr 2015). More specifically, the ab2 sensillar type was selected because the two OSNs housed within it represent both conserved and divergent receptors. *Or59b* (ab2A) is one of the most highly conserved odorant receptor genes across all currently sequenced drosophilids (Guo and Kim 2007). Additionally, previous studies within the Sophophora group have shown conserved electrophysiological responses of the ab2A neuron across species, with the exception of *D. sechellia* which appears to have an extremely diminished number of ab2 sensilla in favor of the ab3 sensillar type (Stensmyr et al. 2003a; Dekker et al. 2006; de Bruyne et al. 2010). In contrast, the odor response profile of the ab2B neuron has been found to be highly variable across species (de Bruyne et al. 2010). The results of this study parallel these earlier findings in the Sophophora group as the ab2B neuron is more divergent than the ab2A neuron (Figs. 2 and 4). Overall the ab2B neuronal responses from the columnar species form a mostly distinct group (90% confidence interval, Fig. 5b), while generalists and *Opuntia* specialists are mixed in both the clustering and phylogenetically corrected PCA. Potentially important chemicals for this separation (based on PC2 loadings) are ethyl hexanoate, furaneol methylether, and ethyl acetate.

The ab3 sensillar type was examined because it has been proposed to be adapted for species specific host volatile detection (de Bruyne et al. 2010; Stokl et al. 2010; Mansourian and Stensmyr 2015). We found high variability in ab3A responses between species (Fig. 3), but no clear association with host type or with phylogeny, for instance *D. melanogaster* responses clustered with several cactophilic species (Fig. 4). Perhaps ab3A does not play as large of a role in host shifts in the *repleta* group as has been shown in the Sophophora group. However, this lack of signal associated with host shift could be a result of only having ab3 results for one representative columnar species (*D. uniseta*), in this study as ab3 sensilla were either not detected (*D. parisiena* and *D. anceps*) or had limited data (*D. nigrospiracula*) in the other columnar specialists. Further studies that focus on columnar species are needed to determine the extent to which ab3 responses have diverged within the *repleta* species group. In the case of the ab3B neuron, the olfactory receptor gene, associated with this neuron in *D. melanogaster*, *Or85b*, is not predicted to be present in *D. mojavensis* (Guo and Kim 2007) and previous recordings of ab3B responses in *D. mojavensis* revealed little to no response to odorants typically diagnostic of this neuronal type in *D. melanogaster* (Crowley-Gall et al. 2016). This study again found little to no ab3B neuron responses in *D. mojavensis* nor in the other cactophilic species examined (Fig. 3). These results suggest that the gene loss (*Or85b*) observed in *D. mojavensis* may be conserved across the *repleta* group and further characterization of additional sensillar subtypes could reveal additional candidate receptors for genomic analysis.

The present results align with previous characterization of the *D. mojavensis* olfactory system. Namely, the results do not identify a shift in sensitivity to a single compound facilitating host shift to columnar cactus, but rather that host specialization in cactophilic flies is likely based on a mixture of odors rather than a single compound and has resulted in a broader altering in response profile (Fig. 5; Date et al. 2013). This work supports the importance of a mixture of cactus odors for host-shifting, as there are no clear associations of single odor sensitivity or specificity differences within the *repleta* group associated with a particular host cactus type. However, clustering of odor response profiles, especially in the ab2B neuron, shows some association with host cactus use which may imply that suites of chemicals contribute to host shifts (Fig. 4). This is supported by the pPCA analyses which showed separation between the columnar specialists and the other two host groups, but with many odorants likely contributing to the variation.

There are a few single odor responses that are noteworthy because they may underlie some routes to specialization. First, the three odorants that seem to be contributing to the differences in ab2B response between the *repleta* species group and *D. melanogaster*, 1-hexanol, (3Z)-Hexenol, and ethyl-3hydroxy butyrate (Fig. 2), which have been associated with fruit volatiles, and (3Z)-hexenol, a green leaf volatile associated with leaf damage (Stensmyr et al. 2003b; Scala et al. 2013). However, these compounds are either found in low abundance or have not previously been identified as components of cactus volatile headspace, making them potentially irrelevant to cactus rot ecology (Flath and Takahashi 1976; Date et al. 2013, 2017; Wright and Setzer 2013b, 2014a). They were included here as compounds diagnostic of specific sensillar subtypes, suggesting that detection of these odorants may not be as necessary for cactophilic flies.

Secondly, *D. leonis* ab2A responses are in different parameter space (Fig. 5a) and a more isolated cluster (Fig. 4a) from other species due to high sensitivity to linalool, linalool oxide and 4-methylphenol (Fig. 2). The latter odorant has previously shown variable neuronal responses on the maxillary palp within *D. mojavensis* populations, and these differences in neuronal response can be linked to variation in odor-induced feeding behavior (Crowley-Gall et al. 2019). However, 4-methylphenol has been found in the volatile headspace of both columnar and *Opuntia* rots (Date et al. 2013, 2017) and was not found to associate with host-type in our analyses, therefore it is unlikely to drive host plant shifts in our system. Interestingly, linalool and linalool oxide have been shown to be major components in the volatile headspace of several *Opuntia* cactus species; however, its presence and abundance can vary based on species and geographic location (Wright and Setzer 2013a, 2014a, b; Date et al. 2017). The strong response to these compounds in *D. leonis* (*Opuntia* specialist) and the lack of response in *D. anceps* and *D. nigrospiracula* (columnar specialists) suggests that a loss of response to *Opuntia* volatile cues in the *anceps* complex may be driving divergence in cactophilic species. This shift in response for *D. leonis* to *Opuntia* volatiles appears to be limited to this specific clade and not host-type. Although linalool and linalool oxide are among the top contributing odors leading to separation of species along PC2, with *D. leonis* being completely separated from *D. anceps* and *D. nigrospiracula* (Fig. 5a), there is not as clear of separation among the rest of the species specializing on *Opuntia* cactus. Though no signal of convergence toward different response rates based on host type were identified in this study, it is possible that larger datasets of species, including more columnar specialists, may reveal a signal that is too weak in this preliminary dataset. Future studies are needed to examine this and other potential processes underlying host shift within the *repleta* species group. For example, future behavioral studies in *D. leonis* could examine whether linalool and linalool oxide act as attractive compounds in host determination for that species.

Finally, much has been done in the recent years to examine volatile headspaces of cacti; however, these studies are limited to specific cactus species or flowering cacti instead of rotting cacti. Future work examining volatile headspace of a wider range of rotting cacti species could better inform our understanding of the divergence, along with its underlying mechanisms, that has occurred between columnar and *Opuntia* specialists. Overall, this study examines sensory neuron sensitivity across the *Drosophila repleta* species group and is a necessary first step in understanding the mechanisms underlying divergence in cactophilic *Drosophila*. More broadly this study provides insight into how variation in the olfactory system can contribute to divergence and host specialization.

## Electronic supplementary material

Below is the link to the electronic supplementary material.

Supplementary Material 1

## Data Availability

No datasets were generated or analysed during the current study.
