# Supplementary material for: Olfactory variation among closely related cactophilic Drosophila species
Source: J Comp Physiol A Neuroethol Sens Neural Behav Physiol. 2025 Jun 12;211(4):445–59. doi: 10.1007/s00359-025-01744-7 (PMC12321934; doi:10.1007/s00359-025-01744-7)
Supplement: Supplementary file 1 — Supplementary Material 1 [file 359_2025_1744_MOESM1_ESM.docx]

**Supplementary Table 1:** Cactus associations for odorant panel used in this study. Table indicates presence of odorant in a chemical analysis of cactus rot. Data is compiled from both natural rots collected in the field and rots induced in the laboratory. Bolded odorants indicate odorants that have been identified as high responders in the target sensilla or have been used to distinguish between sensillar types in previous studies.

| **Odorant** | **Cactus Associations** | **References** |
| --- | --- | --- |
| Linalool | Columnar, *Opuntia* | Flath and Takahasi 1978; Ammar et al. 2012; Date et al. 2013; Wright and Setzer 2013a; Wright and Setzer 2014b; Date et al. 2017 |
| Linalool oxide | *Opuntia* | Wright and Setzer 2013a; Wright and Setzer 2014a; Wright and Setzer 2014b; Date et al. 2017 |
| Nonanal | Columnar, *Opuntia* | Flath and Takahasi 1978; Wright and Setzer 2013b; Wright and Setzer 2014a; Wright and Setzer 2014b; Date et al. 2017 |
| 2-nonanone | Columnar, *Opuntia* | Date et al. 2013; Wright and Setzer 2013b; Wright and Setzer 2014a; Date et al. 2017 |
| 6-methyl-5-hepten-2-one | Columnar, *Opuntia* | Wright and Setzer 2014b, Date et al. 2017 |
| **Acetoin** | Columnar, *Opuntia* | Date et al. 2013 |
| Acetone | Columnar, *Opuntia* | Flath and Takahasi 1978; Date et al. 2013; Date et al. 2017 |
| **Furaneol methylether** |  |  |
| 2-phenethyl acetate | Columnar, *Opuntia* | Date et al. 2013; Date et al. 2017 |
| **Ethyl-3-hydroxybutyrate** | *Opuntia* | Flath and Takahasi 1978; |
| **Ethyl acetate** | *Opuntia* | Flath and Takahasi 1978; |
| **Ethyl butanoate** | Columnar, *Opuntia* | Flath and Takahasi 1978; Date et al. 2013; Date et al. 2017 |
| **Ethyl hexanoate** | *Opuntia* | Flath and Takahasi 1978; |
| Ethyl salicylate | Columnar, *Opuntia* | Date et al. 2017 |
| Hexyl acetate | Columnar, *Opuntia* | Date et al. 2013; Date et al. 2017 |
| **Isopentyl acetate** | Columnar, *Opuntia* | Date et al. 2013; Date et al. 2017 |
| Isopentyl propionate | Columnar, *Opuntia* | Date et al. 2013; Date et al. 2017 |
| **Methyl hexanoate** |  |  |
| **Methyl salicylate** | Columnar, *Opuntia* | Date et al. 2013; Wright and Setzer 2014a; Wright and Setzer 2014b; Date et al. 2017 |
| **Pentyl acetate** | Columnar | Date et al. 2017 |
| Phenethyl propionate | Columnar, *Opuntia* | Date et al. 2013 |
| Prenyl acetate | *Opuntia* | Wright and Setzer 2013a; Wright and Setzer 2014b; Date et al. 2017 |
| 4-ethylguaiacol | Columnar, *Opuntia* | Date et al. 2013; Wright and Setzer 2014a; Date et al. 2017 |
| 4-methylphenol | Columnar. *Opuntia* | Date et al. 2013; Wright and Setzer 2014a; Date et al. 2017 |
| **Acetophenone** | Columnar; *Opuntia* | Flath and Takahasi 1978; Date et al. 2013; Wright and Setzer 2013a; Wright and Setzer 2014b; Date et al. 2017 |
| Benzaldehyde | Columnar, *Opuntia* | Flath and Takahasi 1978; Date et al. 2013; Wright and Setzer 2013a; Wright and Setzer 2014a; Wright and Setzer 2014b; Date et al. 2017 |
| Creosol | Columnar, *Opuntia* | Date et al. 2013; Wright and Setzer 2014a; Date et al. 2017 |
| Guaiacol | Columnar, *Opuntia* | Date et al. 2013; Wright and Setzer 2014a; Date et al. 2017 |
| Methyl eugenol | Columnar | Wright and Setzer 2014a; |
| Phenethyl amine |  |  |
| Phenol | Columnar, Opuntia | Date et al. 2013; Wright and Setzer 2014a; |
| Phenylacetaldehyde | *Opuntia* | Wright and Setzer 2013a; Wright and Setzer 2014b |
| (3Z)-hexenol | Columnar, *Opuntia* | Flath and Takahasi 1978; Wright and Setzer 2013b; Wright and Setzer 2014a; Wright and Setzer 2014b; Date et al. 2017 |
| **1-hexanol** | Columnar, *Opuntia* | Flath and Takahasi 1978; Date et al. 2013; Wright and Setzer 2013a; Wright and Setzer 2013b; Wright and Setzer 2014a; Wright and Setzer 2014b; Date et al. 2017 |
| **1-octen-3-ol** | Columnar, *Opuntia* | Wright and Setzer 2014b, Date et al. 2017 |
| **3-octanol** | Columnar | Date et al. 2017 |
| Ethanol | *Opuntia* | Flath and Takahasi 1978; |
| Isopentyl alcohol | Columnar, *Opuntia* | Date et al. 2013; Date et al. 2017 |
| Isopropyl alcohol | Columnar, *Opuntia* | Flath and Takahasi 1978; Date et al. 2017 |
| Methanol |  |  |
| Acetic acid | Columnar, *Opuntia* | Date et al. 2013; Date et al. 2017 |
| Butyric acid | Columnar, *Opuntia* | Date et al. 2013; Wright and Setzer 2014a; Date et al. 2017 |
| Propionic acid | Columnar | Date et al. 2013 |

**Supplementary Table 2:** Analysis of selection signals in a phylogenetic framework for highest contributing odorants from PCA analyses. The sensillar subtype for each odorant is listed after the odor name (e.g. 1octen3ol3A corresponds with ab3A responses to 1octen3ol). Likelihood ratio tests (LRT) showed multiple rate models (bmm and oum-boxed) were better than single rate models (bm1 and ou1) for two odorants (bolded). The multistate models for each were too similar to distinguish (delta AIC <2), so both are shown. Notes on these two odors are shown below along with their estimated values of theta for the three groups. Each found *Opuntia* and polymorphic to be similar and columnar to be a distinctly different value.

| Odor | AIC bm1 | AIC bmm | AIC ou1 | AIC oum | LRT p value |
| --- | --- | --- | --- | --- | --- |
| 1octen3ol3A | 120.6512 | 124.3998 | 122.0551 | 122.7538 | >0.05 |
| 1hexanol3A | 122.0522 | 125.6689 | 123.6087 | 125.0124 | >0.05 |
| 1octen3ol3B | 80.89546 | 81.70283 | 82.0346 | 84.76894 | >0.05 |
| 1hexanol3B | 84.16398 | 85.5917 | 84.52257 | 87.12846 | >0.05 |
| **furaneolmethylether3B** | 81.83533 | 76.57118 | 83.20543 | 77.59557 | **0.0082** |
| ethyl3hydroxybutyrateA | 121.8824 | 125.7615 | 123.5843 | 123.7726 | >0.05 |
| ethyl3hydroxybutyrateB | 80.05788 | 83.1705 | 81.84067 | 84.59607 | >0.05 |
| linalooloxide2A | 130.4367 | 129.7069 | 134.1424 | 134.0607 | >0.05 |
| **2phenethylacetate2A** | 96.67928 | 92.30981 | 100.03381 | 90.87413 | **0.0014** |
| Linalool2A | 116.9955 | 117.504 | 118.166 | 119.1686 | >0.05 |
| 4-methylphenol2A | 107.5637 | 111.8101 | 116.8622 | 118.2174 | >0.05 |
| furaneolmethylether2B | 93.06534 | 92.6024 | 88.11116 | 92.0243 | >0.05 |
| ethylacetate2B | 95.52016 | 92.96624 | 89.23444 | 92.0145 | >0.05 |
| ethyhexanoate2B | 117.5199 | 102.4355 | 97.56524 | 102.4355 | >0.05 |

| **furaneolmethylether3B** | |  |  |  | **2phenethylacetate2A** | |
| --- | --- | --- | --- | --- | --- | --- |
| OUM thetas | polymorphic 8.112519 |  |  |  | OUM thetas | polymorphic 30.59472 |
|  | *Opuntia* 10.263435 |  |  |  |  | *Opuntia* 25.65786 |
|  | columnar 46.147569 |  |  |  |  | columnar 62.60425 |
| notes | multistate, but similar to brownian motion (delta AIC <2) | | | | notes | multistate, but similar to brownian motion (delta AIC <2) |
